# Computational analysis of genetic network involved in pancreatic cancer in human

**DOI:** 10.1186/1471-2105-12-S11-A11

**Published:** 2011-11-21

**Authors:** Mrinal Mishra, Ambuj Kumar

**Affiliations:** 1 School of Bio Sciences and Technology, VIT University, Vellore, India

## 

The poster is based on the in silico identification and analysis of the variation in the gene network related to the pancreatic cancer. Pancreatic cancer has been a major cause of death in Asia and European countries. This cancer is not easily detectable in its initial stages and at the later stages it becomes very hard to cure this disease. So the in-depth understanding of the variation in the genetic pathway of this disease is very important. We selected 5 candidate genes from various published journals which are involved in the pancreatic cancer pathway (KRAS, CDKN2A, MADH4, TP53 and ARMET) in generating their interaction network using Agilent literature search plugin in cytoscape. The organic layout of interaction revealed the cross interaction between these genes and the other neighbour genes. Merging the expression profile data of the pancreatic cancer to the parent network helped us in understanding the variation of the network in the diseased state. Using the merged profile network we found out the importance of the KRAS & CDKN2A gene interaction with other 21 neighbour genes among which PIK3CA and TP53 interactions were showing major variation on their expression pattern. This study reveals the importance of change in expression level of candidate genes(KRAS,CDKN2A and TP53) in causing pancreatic cancer. The results obtained in our study will be very much useful in detecting the disease in its initial stages and in finding the cure.

